# Stability, optimum ultrasonication, and thermal and electrical conductivity estimation in low concentrations of Al_12_Mg_17_ nanofluid by dynamic light scattering and beam displacement method

**DOI:** 10.1038/s41598-023-40844-9

**Published:** 2023-08-22

**Authors:** Soroush Javadipour, Ali Shokuhfar, Zeinab Heidary, Mohammad Amin Amiri Roshkhar, Keyvan Homayouni, Fatemeh Rezaei, Ashkan Zolriasatein, Shahrokh Shahhosseini, Alimorad Rashidi, S. M. Mahdi Khamoushi

**Affiliations:** 1 Faculty of Materials Science and Engineering, K. N. Toosi University of Technology, 15 Pardis St., Tehran, 1991943344 Iran; 2 Department of Mechanical Engineering, K. N. Toosi University of Technology, 15 Pardis St., Tehran, 1999143344 Iran; 3 Laser and Plasma Research Institute, Shahid Beheshti University, Tehran, Iran; 4 Department of Petroleum Engineering, Science and Research Branch, Islamic Azad University, Tehran, Iran; 5 Department of Physics, K. N. Toosi University of Technology, Tehran, 15875-4416 Iran; 6 Non-Metallic Materials Research Department, Niroo Research Institute, Tehran, Iran; 7 Department of Chemical Engineering, Iran University of Science and Technology, Tehran, Iran; 8 Nanotechnology Research Center, Research Institute of Petroleum Industry IR, Tehran, Iran

**Keywords:** Energy transfer, Computational nanotechnology, Optical spectroscopy, Nanophotonics and plasmonics, Laser material processing, Optical sensors

## Abstract

The thermal conductivity and stability of nanofluids pose challenges for their use as coolants in thermal applications. The present study investigates the heat transfer coefficient (HTC) of an Al_12_Mg_17_ nanofluid through the utilization of a novel beam displacement method. The study also examines the nanofluid's stability, particle size distribution (PSD), TEM micrograph, and electrical conductivity. From three distinct categories of surfactants, a particular surfactant (CTAB) was chosen to disperse Al_12_Mg_17_ nanoparticles in DI water, and subsequently, a two-step method was employed to generate the nanofluid. Dispersion stability is visually monitored and quantified with a zeta potential test. HTC and PSD are measured using optical setups. To evaluate the results, the HTC obtained from the beam displacement method is compared with that of the KD2 Pro apparatus, and the PSD findings are analyzed through TEM micrographs. The results show that a 0.16 vol.% CTAB is the maximum stability for 0.025 vol.% Al_12_Mg_17_ nanofluid properly. The optimum ultrasonication period is 2 h, yielding a peak PSD of 154 nm. Increasing nanoparticle concentration enhances HTC up to 40% compared to the base fluid at 0.05 vol.%. Electrical conductivity increases linearly from 155 to 188 μ${\rm S}/\mathrm{cm}$ with nanoparticle concentration. Optical methods for measuring HTC in nanofluids offer the advantage of early results, prior to bulk motion. Thus, the application of nanofluids in thermal systems necessitates the development of optical techniques to improve accuracy.

## Introduction

A nanofluid is a heterogeneous mixture of a base fluid and nanoparticles which can be utilized in a broad range of thermal applications in both industry^1^ and medicine^2^, including but not limited to solar collectors^3^, vehicle radiators^4^, and electronic cooling^5^. Due to their substantial role in transferring heat, nanofluids can bring efficiency to the system performance, which makes them fascinating area of study for engineers. The differences The differences in thermal conductivity among nanofluids have already been studied^6^. However, it is imperative to characterize thermal and electrical properties of nanofluids as well as their stability and PSD in order to apply them in the industry.

Regarding thermal characterization of nanofluids, scientists use different methods to determine HTC, such as the transient methods, three omega^7^, temperature oscillation^8^, steady state parallel plate^9^, thermal comparator^10^, and optical methods each of which has a different criteria for determination. For instance, the transient hot-wire (THW)^11^ and transient plane source (TPS)^12^ are two examples of transient methods, which are based on monitoring the temperature of heat source and time response after their exposure to an electrical pulse^13^. Also, steady state methods take advantage of thermocouples and it is important to keep the temperature reading discrepancies to a minimum when the thermocouples are at the same temperature^10^. In addition, in thermal comparator, evaluation of the sample's conductivity needs only one point of contact^10^. However, optical methods, which are also used to determine HTC, are based on the interaction between light and fluid.

Generally, a number of optical methods, such as the laser flash analysis (LFA) technique^14^, are used to measure the HTC of nanofluids. Moreover, there are other optical methods, including beam deflection methods^15^ and hot-wire laser beam displacement techniques that rely on the temperature-dependent deflection angle in nanofluids^16^. The hot-wire laser beam displacement method can assess the HTC and thermal diffusivity of nanofluids^16^. Generally, beam displacement method is based on changing the reflecting index by temperature variations so that HTC and thermal diffusivity of nanofluids increase with an increase in volume fraction^16,17^.

Several research groups have reported the variations of the thermal conductivity of different nanofluids, versus the type, size, and the base fluid. Furthermore, different researchers have employed a variety of nanoparticles, including single-element, single-element oxide, multi-element oxides, metal carbides, metal nitrides, and carbon base nanoparticles. These nanoparticles can be dissolved in various liquids, such as water, ethanol, EG, oil, and refrigerants^18–20^. For instance, Paul et al.^20^ mechanically alloyed Al_95_Zn_05_ before using a two-step process to disperse the nanoparticle in ethylene glycol. According to the thermal conductivity characterization, 0.10 vol.% dispersion of nanoparticles resulted in a 16% increase in HTC relative to the base fluid. In our last work, we explored the thermal and electrical conductivity of Al_2_O_3_-ZnO-CNT nanoparticles dispersed in DI water^21^. Also, we investigated the PSD of Al_2_O_3_–ZnO nanofluid versus time in order to study the stability of the nanofluid. It was concluded that by adding carbon nanotubes to Al_2_O_3_–ZnO nanofluid and forming 0.05 wt.% hybrid nanofluid, the HTC was enhanced by 30% in comparison with DI water. In addition, an experimental study on the thermal conductivity of Cu_5_Zn_8_ nanoparticles dispersed in an oil-based fluid was conducted by Farbod et al.^22^. Their result demonstrated that, in comparison to the base fluid, oil-based nanofluids with various concentrations of Cu_5_Zn_8_ nanoparticles had better thermal conductivity. It should be mentioned that intermetallic materials are employed by researchers as nanofluids, like Ti–6Al–4V^23^, Al_95_Zn_05_^20^, Cu_5_Zn_8_^22^, and NiAl intermetallic nano-powders^24^. In addition, Al_12_Mg_17_, which we used in our study, is another intermetallic substance that has been the subject of a few studies^25^.

On the other hand, many research groups have studied the modeling of thermal conductivity. For instance, the study conducted by Lal Kundan and Soumya Suddha Mallick referred to the modeling of thermal conductivity in alumina-water nanofluids. To that end, the accuracy of seven existing theoretical and empirical models for the thermal conductivity of nanofluids was assessed by comparing predicted and experimental data for a wide range of test conditions. The results indicated that the current models exhibit inaccuracies, with over or under-predictions ranging from 2.34 to 58%^26^.

The thermal conductivity of nanofluids is influenced by a range of parameters, including the settling rate of nanoparticles, growth of nanoclusters, morphology and stability of clusters, Brownian motion of the nanoparticles, size distribution, temperature, and liquid layering^27^. The presence of nanoclusters within nanofluids results in better thermal conductivity. Therefore, it is essential to investigate the effect of nanoclusters on nanofluids. The fundamental aspects related to the formation of clusters during aggregation encompass three main factors: the movement of nanoparticles that facilitate their contact, the probability of nanoparticles combining and expanding into clusters, and the mobility or dynamics of the clusters or aggregates^28^. The perikinetic aggregation as well as the Brownian motion within the cluster–cluster aggregation play a key role in the growth of a cluster^29^. Despite the fact that nanoclusters enhance heat transfer, aggregation leads to precipitation, which makes investigating the stability of nanofluids highly challenging.

The relationship between ultrasonication time and the size of the nanoparticles in nanofluids is an essential issue in the evaluation of nanofluid performance. Sonication is a physical technique that can be applied to improve the stability of nanofluids by rupturing the attractional force of the nanoparticles in order to reduce their size^30^. Dynamic light scattering (DLS) is one of the most accessible and practical methods to estimate PSD^31^. For instance, Poli et al.^32^ studied the relationship between the particle size of SAz-1 and SWy-1 montmorillonite and the ultrasonication period. According to their results, 60 min of sonication led SAz-1 to exhibit a relatively broad PSD with a hydrodynamic diameter of 283 nm. Also, the same sonication period led SWy-1 to display a bimodal distribution of particles at 140 and 454 nm. Using DLS, Afzal et al.^33^ reviewed the impact of ultrasonication period on the average particle size of various nanofluids. According to their observations, lengthening the sonication process reduces the particle size, improves dispersion, and increases stability. Furthermore, the optimum ultrasonication period was accomplished, resulting in the highest performance. They also discovered that ultrasonication periods longer than the optimum do not improve the stability.

In this study, not only did we investigated the physical properties of Al_12_Mg_17_ nanofluid, including stability, optimum ultrasonication period, and electrical conductivity, but also assessed the HTC, using an innovative beam displacement method. This method has the novelty of focusing on the complex effect of nanofluid’s thermal properties on the light beam deviation, using an image processing technique. As part of the novelty of the present research, CCD detectors are used instead of PSD detectors for beam displacement detection, since virtual methods may not be as reliable as intuitive methods. In the current study a two-step method, is used to prepare nanofluids and the zeta potential test and sediment observation are then employed to consider the effect of variation in type and vol.% of surfactant on the stability of the Al_12_Mg_17_ nanofluids. Nanofluids exhibit complicated time-dependent behavior after ultrasonication which can be attributed to their size and morphology. There is also a lack of research that evaluates the variations in PSD versus the ultrasonication period. In this regard, the DLS method is exploited to analyze the PSD of the nanofluid versus the ultrasonication period. Moreover, transmission electron microscopy (TEM) is employed to examine the size and morphology of the Al_12_Mg_17_ nanoparticles and analyze the DLS findings. Besides, the HTC outcomes from the beam displacement method are compared with the nanofluid’s HTC measured by the KD2 Pro apparatus. It is shown that the Al_12_Mg_17_ nanoparticles in DI water as the base fluid, stabilized by the surfactant and ultrasonication, significantly affect the thermal properties of the nanofluid.

## Materials and methods

### Preparation method

The present study utilizes Al_12_Mg_17_ nanoparticles with an average particle size of 24 nm, obtained through a 20-h milling process. In this regard, an alloy with a nominal composition of Al_41.4_Mg_58.6_ (γ-Al_12_Mg_17_) was produced in an electrical resistance melting furnace using commercially pure Al (99.7%) and Mg (99.9%) in a protective argon atmosphere. The cast ingot's core was sliced into small samples, which were vacuum sealed in quartz tubes. After conducting homogenization heat treatments in an electrical furnace, which was set at 400 °C degrees for varying lengths of time, the tubes were quenched in water. Two prevalent techniques for fabricating complex metal alloy γ-Al_12_Mg_17_ nanoparticles are the planetary ball mill and SPEX mill^25,34^. producing Al_12_Mg_17_ powder was conducted in an argon-filled planetary high-energy ball mill (Fritsch—P6). Crushed and pulverized material for milling experiments passed through a 140-mesh sieve. The powders were then charged in milling vials with 10-mm steel balls for a 10:1 ball-to-powder mass ratio (BPR). The powders were milled for up to 20 h at an intensity of 250 rpm. After 20 h of ball milling, it was discovered that particles with an average size of 24 nm and crystallites of 16 nm were produced as a result of the procedure^35^.

A two-step process was used to disperse the nanoparticles in the base fluid in order to create Al_12_Mg_17_ nanofluids. The two-step procedure involves producing nanoparticles separately first, and then, dispersing the created nanoparticles in the base fluid using a variety of physical treatment methods, such as utilizing a stirrer, an ultrasonic bath, and an ultrasonic disruptor^36^.

In this study, the Al_12_Mg_17_ nanofluids with varying volume concentrations were created by diluting the concentrated suspensions. Table 1 summarizes the details of the preparation procedure. The study has employed three discrete classifications of surfactants, (anionic, nonionic, and cationic), these surfactants were utilized to determine the compatibility of Al_12_Mg_17_ nanoparticle surfaces with different surfactant types. Here, PVA (polyvinyl alcohol) as a nonionic surfactant, SDS (sodium dodecyl sulfate) as an anionic surfactant, and CTAB (cetyltrimethylammonium bromide) as cationic surfactants were used to produce the nanofluids. Table 2 demonstrates the details of the surfactant utilized for producing nanofluids. Moreover, five different concentrations of CTAB surfactant including 0.02, 0.04, 0.06, 0.08, and 0.1 vol.% were added into 100 ml of DI water.

**Table 1:** Details of the methods utilized for producing nanofluids by two-step technique.

Preparation process	Process details
Stirrer	Revolution speed: 900 rpm Revolution time: 15 min
Ultrasonic disruptor	Sonication time: Variable (15, 30, 45, 60, 75, 90, 105, 120, 135, 150, 165, 180) Frequency: 60 kHz Max. sonicating power: 250 W
Ultrasonic bath	Sonication time: 30 min Frequency: 40 kHz Max. sonicating power: 150 W

**Table 2:** Details of the surfactant utilized for producing nanofluids.

Type of surfactant	Chemical formula	Properties
SDS	C_12_H_25_NaSO_4_	Anionic surfactant^37^, white or cream-colored solid,
PVA	[CH_2_CH(OH)]_n_	nonionic surfactant^38^, good chemical resistance, high hydrophilicity, nontoxic^39^, dissolvable in water
CTAB	C_19_H_42_BrN	Cationic surfactant^37^, white powder, dissolvable in water

### Testing procedure

**Figure 1:**
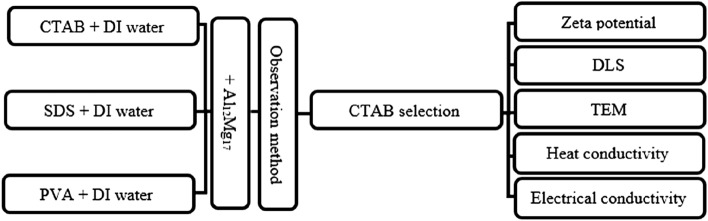
Different steps of the testing procedure: preparation and characterization of the nanofluid.

First, 0.1 vol.% of PVA, SDS, and CTAB were individually dissolved in DI water. Then, 0.05 vol.% of Al_12_Mg_17_ nanoparticles were added to each solvent during its stir. Under the conditions described in Table 1, both of the ultrasonic disruptor and ultrasonic bath were employed to disperse nanoparticle clusters into the nanofluids. When using the observational approach to assess the stability of nanofluids containing various surfactants, the nanofluid including CTAB showed appropriate stability. As a result, CTAB was used as the surfactant in all subsequent tests, and its optimal amount was chosen by comparing the zeta potential values at various concentrations. After selecting the optimal surfactant concentration, the optimum ultrasonication period was determined by utilizing the DLS technique to follow the PSD. The DLS results in the current study were validated using TEM microscopy images. Final step of the test protocol involved measuring of the thermal and electrical conductivities for different mass fractions of Al_12_Mg_17_ nanofluids. Figure 1 depicts the procedure's flowchart.

### Stability measurements

Various techniques have been used by researchers to evaluate the stability of the nanofluids, including; observational methods^40^, measurements of UV light absorbance spectra^41^, pH values^42^, and zeta potential analysis^43^. In this study, the stability of the Al_12_Mg_17_ nanofluid is assessed utilizing the observation method, and the zeta potential test. An observation method is based on taking pictures of a sample which contains generated nanofluid in a glass tube as time passes. It should be mentioned that it is possible to compare the collected images together and follow the sedimentation rate of the nanofluid^44^. Zeta potential testing is another method for evaluating the stability of nanofluids which involves measuring each sample of the Al_12_Mg_17_ nanofluid's zeta potential using a ZETA-check apparatus (Particle Metrix, Germany). Zeta potential can be calculated using the Henry equation by employing the electrophoretic mobility of particles in suspension^45^. In this research, the reported zeta potential values are the averages magnitudes obtained from three replicates of each sample.

### PSD measurements

The DLS method is a non-imaging method for figuring out the average size and the size distribution of particles in suspension or polymers in solution. The size of particles between one nanometer and several micrometers can be determined using this method. A coherent light source, such as a laser disperses in diverse directions when it strikes a solution medium that contains particles. This kind of scattering for nanoparticles is called Rayleigh scattering, since the diameter of nanoparticles is significantly smaller than the wavelength of the laser (i.e., 632.8 nm), while for larger particles it follows the patterns of Mie scattering and Fraunhofer diffraction, respectively. The intensity of the scattered light fluctuates over time as a result of the Brownian motion of the particles inside of the solution. It should be noted that the frequency of variations in the intensity of scattered light by the larger particles is lower because of their slower rate of movement in the solution, and vice versa. A typical graph is produced by computing the autocorrelation function (ACF) of the intensity, causing its decay curve depend on the particle size. Furthermore, the size and morphology of the Al_12_Mg_17_ nanoparticles in the nanofluid are examined, and as well as the validity of the DLS findings are determined using transmission electron microscopy (TEM, Zeiss EM-10C).

#### Theory of the DLS method

Scattering is a common phenomenon that occurs together with Absorption. A beam of light that is passing through the medium might lose energy due to absorption and scattering. When light travels through a material that is entirely homogeneous, there is no scattering of the light. Only the presence of inhomogeneities results in scattering. In a fluid, the true scattering that occurs is caused by statistical variations in the arrangement of the molecules or particles. It is feasible to investigate the scattering if the particles in question are sufficiently separated from one another^46^. In fact, Scientists use a computation called Fraunhofer Diffraction if the particles are large in comparison to the wavelength of the light. The Mie scattering calculation is used when the particle size is either comparable to or less than the wavelength of the light^47^.

Mie scattering is generally applied when electromagnetic energy is scattered out of sphere particles, the size of which is equivalent to or less than the light wavelength^48^.

The DLS technique involves the employment of a coherent laser beam to excite a sample, including Brownian-moving particles in a solution. Particles in the path of the laser beam scatter light in different directions. The detector records the variations in the light intensity scattered over time that occur at a specific angle with respect to the incident beam propagation direction. The light scattered from moving particles can provide information about their movement patterns. The diffusion coefficient increases as frequencies of fluctuations in the scattering intensity enhance, and vice versa.

The relationship between the particle size and its thermal movement pattern is the foundation of the DLS approach. The Stokes–Einstein equation presents the definition of this relationship^49^ as:

1 $$D=\frac{{K}_{B}T}{3\pi \eta d}$$

here $D$ is the diffusion coefficient, $d$ is the particle’s hydrodynamic diameter, $\eta$ is the viscosity of the solution, and ${k}_{B}T$ is thermal energy. This equation expresses that a smaller particle size ($d$) causes a faster movement in the solution and a larger diffusion coefficient and vice versa.

In addition, calculating the ACF of the detector's signal is necessary to derive the quantitative relationship between the intensity and diffusion coefficient. This can be typically accomplished with the correlator device. The ACF fitting function displays an exponential decaying relation as Eq. (2)^49^:

2 $$G\left(\tau \right)=\mathrm{exp}(-\Gamma \tau )$$

where $\tau$ is decay time, $G\left(\tau \right)$ is the ACF function, and $\Gamma$ is the decay rate of this function. The decay rate and the diffusion coefficient of the sample particles in the solution are related below^49^:

3 $$D= \frac{{q}^{2}}{\Gamma }$$

where $q$ is the scattering vector which is defined as^49^:

4 $$q=\frac{4\pi {n}_{0}}{{\lambda }_{0}} \mathrm{sin}(\frac{\theta }{2})$$

where ${\lambda }_{0}$ is the laser’s wavelength, ${n}_{0}$ is the solution's refractive index, and $\theta$ is the observation angle of scattering. The diffusion coefficient can be obtained by replacing $q$ from Eq. (4) in Eq. (3), and the hydrodynamic diameter of the sample particles can be calculated from Eq. (1).

#### Experimental set-up for PSD measurements

**Figure 2:**
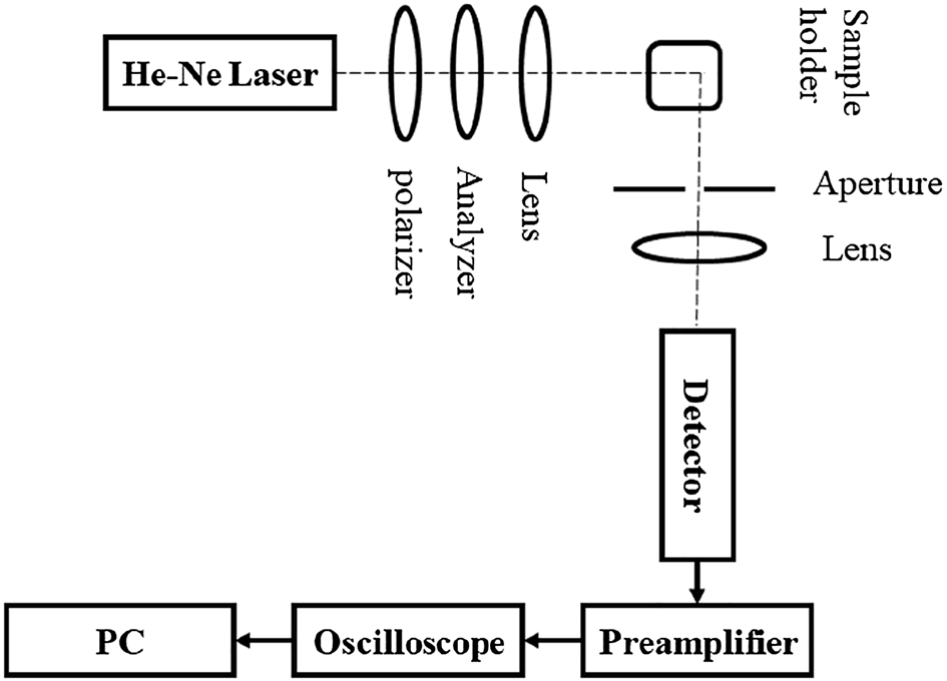
A schematic view of the DLS set-up utilized for PSD measurements.

In this paper, nanoparticles are excited using a 632.8 nm He–Ne laser as shown in the schematic of the experimental set-up in Fig. 2. The laser beam passes through a vertical polarization after passing through a linear polarizer. After traveling through the second polarizer for intensity adjustment, the laser beam is focused by the lens on the sample cuvette containing nanoparticles suspended in solution. Another lens with a 75 mm focal distance is used to focus the scattered light at an angle of 90° with respect to the direction of beam propagation. A PMT detector was employed to detect the scattering. An aperture with a diameter of 1 mm is positioned in front of the collector lens to ensure the formation of an appropriate coherence region on the detector surface. Finally, the signal received from the PMT after pre-amplifying is retrieved with the aid of a digital oscilloscope at a frequency of 100 kHz and then, forwarded into the computer to perform various studies such as computing the ACF.

### Thermal conductivity measurements

In this research, the determination of HTC is performed by the usage of a novel beam displacement technique. Here, by applying a heat source to a nanofluid, changes in temperature affect the fluid's density and, as a result, its refractive index. When the beam travels through a path near the cylindrical heater, these variations lead to a beam deviation. The beam displacement method presented in the current research has three main steps: experimental evaluation of the beam displacement, numerical analysis of the temperature variations in nanofluid, and HTC calculation. In the first step, an optical set-up is designed to record the beam displacement after the deviation of the beam due to applying a thermal shock into the nanofluid. In the second step, a numerical simulation is conducted to calculate the temperature variations through a line corresponding to the beam path. In the third step, a trial-and-error loop is utilized to compare the experimental results with the numerical simulations which obtains the HTC of nanofluid. More details for each step are provided in the following sections.

#### Experimental evaluation of the beam displacement

**Figure 3:**
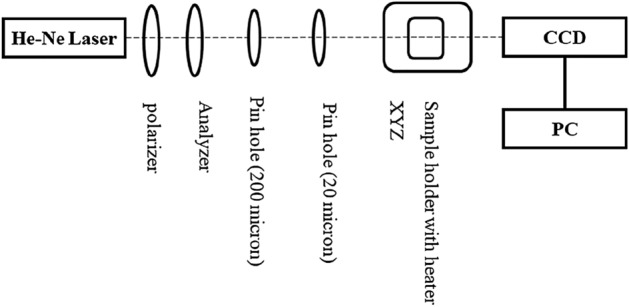
A Schematic of the experimental set-up designed for beam displacement measurement.

Figure 3 depicts the experimental layout used for the beam displacement measurements. A He–Ne laser with a 2-mW output at a wavelength of 632 nm is employed in the experiments. The polarizer and analyzer regulate the intensity of the laser light. A lens with a 50 mm focal length concentrates the laser beam on the samples. Additionally, two pinholes with hole widths of 50 and 20 microns are placed respectively for guiding the laser beam, and alignment is accomplished by the XYZ-stage, and the heater is secured by a specifically built cap on the cuvette. A CCD detector detects the beam displacement, and the outputs are sent to the PC. As part of the novelty of the present research, since virtual methods may not be as reliable as intuitive methods, CCD detectors are used instead of PSD detectors.

In order to evaluate the thermal conductivity magnitudes, Ali et al.^16^ proposed the heat transport equations relevant to the beam displacement method as below:

5 $${\delta }_{T}= \frac{w- W+ {n}_{0} {L}_{air}}{{n}_{0}}\frac{dn}{dT}{\int }_{-W}^{W}\frac{D}{\sqrt{{D}^{2}+{Z}^{2}}}\frac{dT}{d\rho }dz.$$

In this equation, ${\delta }_{T}$ is the beam displacement, $w$ is the half width of the cuvette, $W$ is the inner size of the cuvette within which the probe beam deflected, ${L}_{air}$ is the distance between the cuvette and CCD detector, $n$ is the nanofluid’s refractive index which depends on temperature, ${n}_{0}$ is the normal value, $\rho$ is the radial coordinate, $z$ is the spatial direction parallel to the original beam path, $D$ is the distance between the center of heater and probe beam, $Z$ is the adjacent side of a triangle with $\rho$ as hypotenuse and $D$ as opposite side, and $dT/d\rho$ is the temperature distribution which is obtained by a numerical simulation using finite element method (FEM). In addition, ${\delta }_{T}$ is extracted through an image processing method. The CCD detector is employed for the image processing, and the location of the brightest spot is found by Python software. It should be noted that the initial location of the brightest spot on the screen is recorded before the thermal shock was administered. After 5 s of exposure into the thermal shock, the beam deflection is recorded by measuring the displacement of the brightest spot. The fluid surrounding the heater’s surface will experience greater temperature changes, hence, the beam must travel only a very small distance from the heater. In the current experiment, the heater's surface is 100 microns away from the place where the beam passed. Furthermore, controlling the light intensity is another crucial factor. If fewer beams pass close to the heater, the beam displacement is easier to be detected. Here, a CMOS sensor captures the beam-spot intensity, and the Python OpenCV package is then applied to analyze the data. The sampling frames came into the computational process to measure the beam displacement during experiments. Moreover, the intensity profile is fitted using a Gaussian function to increase measurement accuracy with a resolution of 100 nm.

#### Numerical analysis of the temperature variations by FEM

**Figure 4:**
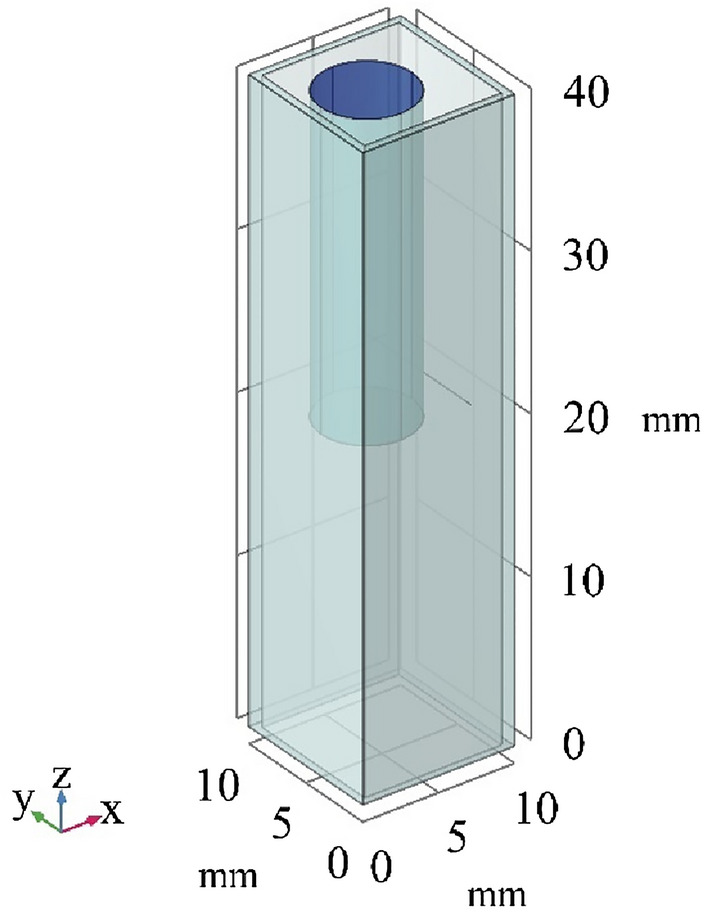
A schematic view of the geometry applied for the numerical simulations.

The second step of the beam displacement method is evaluation by simulating the thermal variations on the beam path line. The computational study of the temperature evolutions in the numerical model is performed using finite element method. As depicted in Fig. 4, a 3D geometry of the cuvette containing fluid and cylindrical heater was modeled in accordance with the actual dimensions of the experimental set-up. To define the material properties, the cuvette and cylindrical heater were assumed to be solid bodies with specified densities, thermal conductivities, and heat capacities (Table 2). Corresponding material constants for the fluid are presented in Table 3 and were employed for simulating the fluid’s thermal behavior in the present study. To ascertain the fluid's HTC, it was required to run simulations for a variety of HTC values in order to compare the results with the outputs of the beam displacement experiments.

**Table 3:** Material properties considered for different parts of the numerical model.

Region	Density ($\frac{\mathrm{kg}}{{\mathrm{m}}^{3}}$)	HTC ($\frac{\mathrm{W}}{\mathrm{m K}}$)	Heat capacity ($\frac{\mathrm{J}}{\mathrm{kg K}}$)
Cuvette	2210	0.8	730
Cylindrical heater	7000	50	420
Fluid	1005	–	4250

In this paper, the thermal insulation conditions were applied for the exterior boundaries and the heater body is assumed to be a heat source with a heat generation power of 90 $\mathrm{MW}/{\mathrm{m}}^{3}$. As shown in Fig. 4, to determine the temperature variations, a 150 microns line from the heater surface was defined to correspond to the trajectory of the beam in the tests. All components were assumed to have a starting temperature of 298 K, which matches the temperature of the experiment’s environment. Furthermore, the heat transfer equation was applied to the physical problem at the interface. A time-dependent problem is solved to obtain the temperature evolutions throughout all regions for 5 s.

#### HTC calculation

The calculation of HTC in this study involves comparing the beam displacements obtained from experimental results with those obtained from numerical simulations. As mentioned before, an optical setup is initially designed to capture the beam displacement resulting from the applied thermal shock to the nanofluid. Subsequently, numerical simulations are performed using the finite element method to calculate the temperature variations along the beam path.

**Figure 5:**
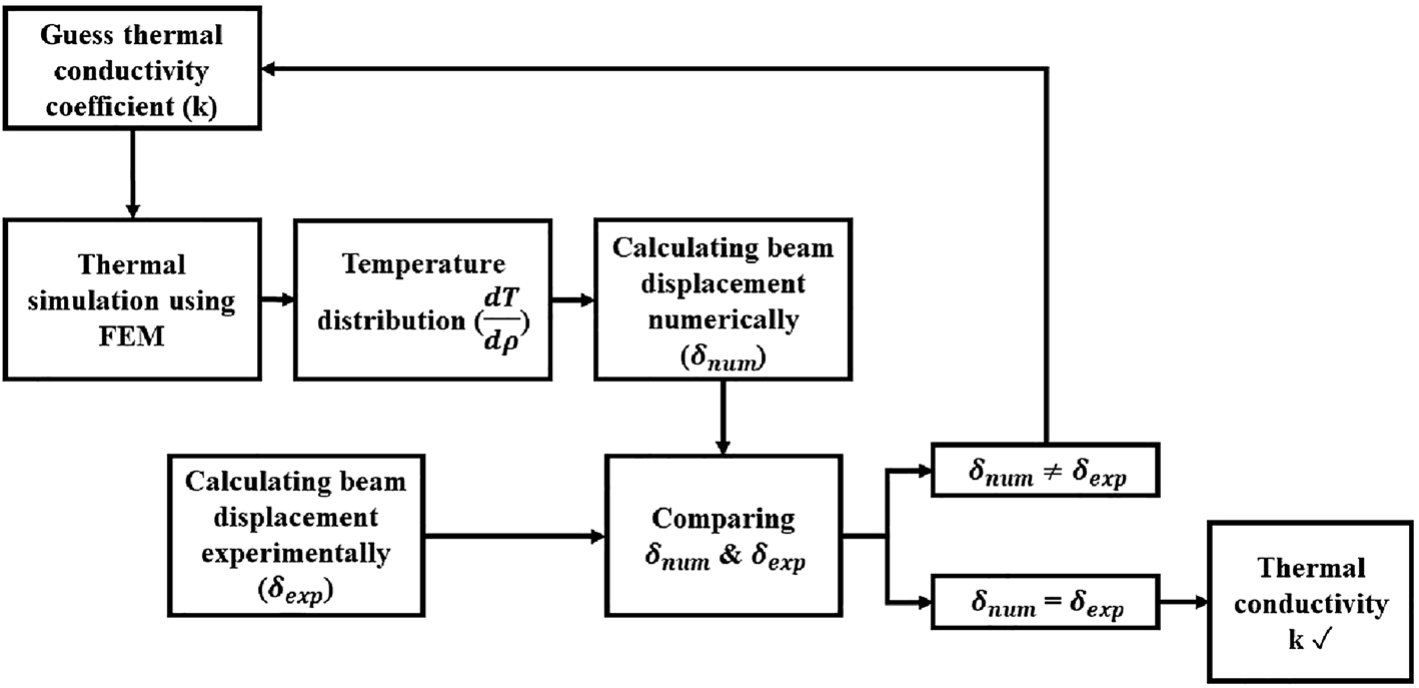
A schematic diagram outlining the procedures for the calculation of the HTC using beam displacement measurements and FE simulations.

To determine the HTC of the nanofluid, a trial-and-error loop is implemented. Starting with an initial guess for the HTC, which is assumed to be the HTC of the base fluid, the thermal variations through the nanofluid are simulated. A MATLAB code solved the right-hand side of Eq. (5) using the temperature profiles obtained from the numerical simulations. The left-hand side of Eq. (5) represents the experimental beam displacement, which is used to iteratively refine the estimation of the HTC within the trial-and-error loop. The iterative process, guided by the comparison between the beam displacement obtained from experimental results and numerical simulations, facilitates the convergence towards an accurate determination of the HTC for the nanofluid. This approach ensures a precise calculation of the HTC, as depicted in the flowchart shown in Fig. 5.

#### Comparison of HTC measurements

A KD2 Pro apparatus was used at 25 °C (Decagon Devices Inc., Pullman, WA, USA) to compare the HTC of the nanofluids with those obtained by the beam displacement method. The transient line heat source method underlies how the KD2 Pro analyzer operates. Before usage, the device was calibrated employing a standard sample of glycol. The measurements were performed after passing enough time for establishing the temperature equilibrium. A little amount of heat is applied to the needle by the KS-1 sensor, which aids in preventing free convection in liquid samples. Moreover, due to the sedimentation, the average HTC was measured three hours after preparation by repeating the tests five times.

### Electrical conductivity measurements

Electrical conductivity was estimated using the PCT-407 apparatus with a measuring range of 0–200 mS, and a 2% FS accuracy. Moreover, there is a nominal cell electrode in the PCT-407 device. The device was automatically calibrated using a calibration solution prior to conducting measurements. It should be noted that due to the sedimentation, the average electrical conductivity was determined six hours after preparation by repeating the tests five times.

## Result and discussion

### The stability results

#### Observation method

**Figure 6:**
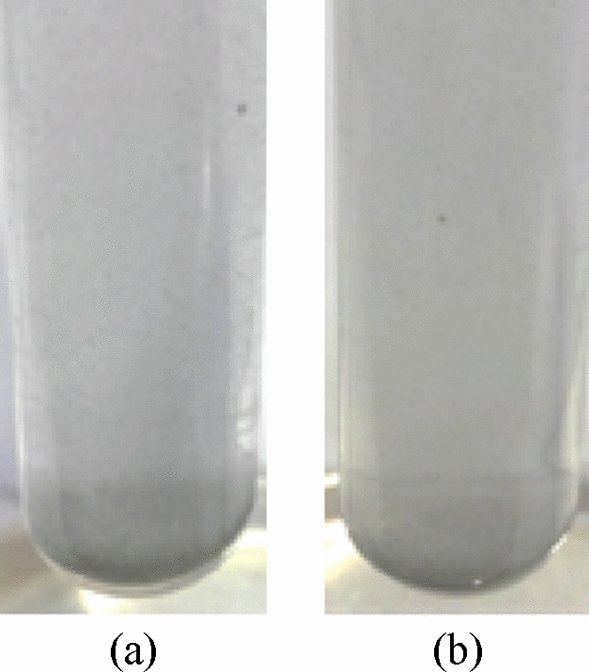
Al_12_Mg_17_ nanofluids 30 min after preparation with (**a**) PVA, and (**b**) SDS.

The observation method is used to investigate the stability of nanofluids by adding three surfactants, including PVA, SDS, and CTAB. Figure 6a,b show the sedimentation observations of 0.025 vol.% Al_12_Mg_17_ nanofluids which were prepared after 30 min of sonication with PVA and SDS as surfactants. As demonstrated in Fig. 6, using PVA and SDS made the suspension unstable. In fact, both PVA and SDS similarly reduce the stability of the 0.025 vol.% Al_12_Mg_17_ nanofluid and made nanoparticles first agglomerate and then sediment only relatively shortly after the preparation of nanofluids.

Contradictorily to PVA and SDS, CTAB demonstrated the proper stability. Therefore, CTAB was used as a surfactant to prepare Al_12_Mg_17_ nanofluids at various concentrations.

Generally, surfactants prefer to be found at the interface between the nanoparticles and fluids because they create a sort of continuity between the two phases^50^. An interfacial layer is formed around the nanoparticles as a result of the accurate amount of surfactants delivered into the nanofluid which is absorbed on the interface^51^. Studies have shown that adsorption is influenced by the characteristics of the solid substrate, the solvent, and the type of the surfactant that is used^52^. It should be mentioned that SDS is an anionic surfactant^53^, CTAB is a cationic surfactant^54^, and PVA is a non-ionic polymer compound^55^. The compatibility of the surfactant depends on the surface charge of the Al_12_Mg_17_ nanoparticles.

When Al_12_Mg_17_ nanoparticles disperse in SDS + DI water solution, no adsorption happens between the surfactant and nanoparticles and instead the nanoparticles start to agglomerate. This can be explained by the presence of repulsive inter molecule forces among SDS and Al_12_Mg_17_ nanoparticles, which result from the negative charges on the surface of Al_12_Mg_17_ nanoparticles. the PVA chains are comprised of a specific quantity of acetate groups (14%), which are responsible for conferring a negative charge to the polymer molecules. Since Al_12_Mg_17_ nanoparticles are negatively charged on the surface, the negative charge that is conferred by even a small group of acetates behaves like an obstacle, hampering the interaction between Al_12_Mg_17_ nanoparticles and PVA. On the other hand, the desired adsorption among CTAB, as the cationic surfactant, and Al_12_Mg_17_ nanoparticles, prevents the Al_12_Mg_17_ nanoparticles from agglomeration.

#### Zeta potential analysis

The zeta potentials of the 0.025 vol.% Al_12_Mg_17_ nanofluid, utilizing 0.1 vol.% PVA, SDS, and CTAB as surfactants, are + 3.8, − 20.3, and 47.1 mV, respectively. Based on these results, CTAB has been identified as the most effective surfactant for ensuring the stability of the Al_12_Mg_17_ nanofluid.

**Figure 7:**
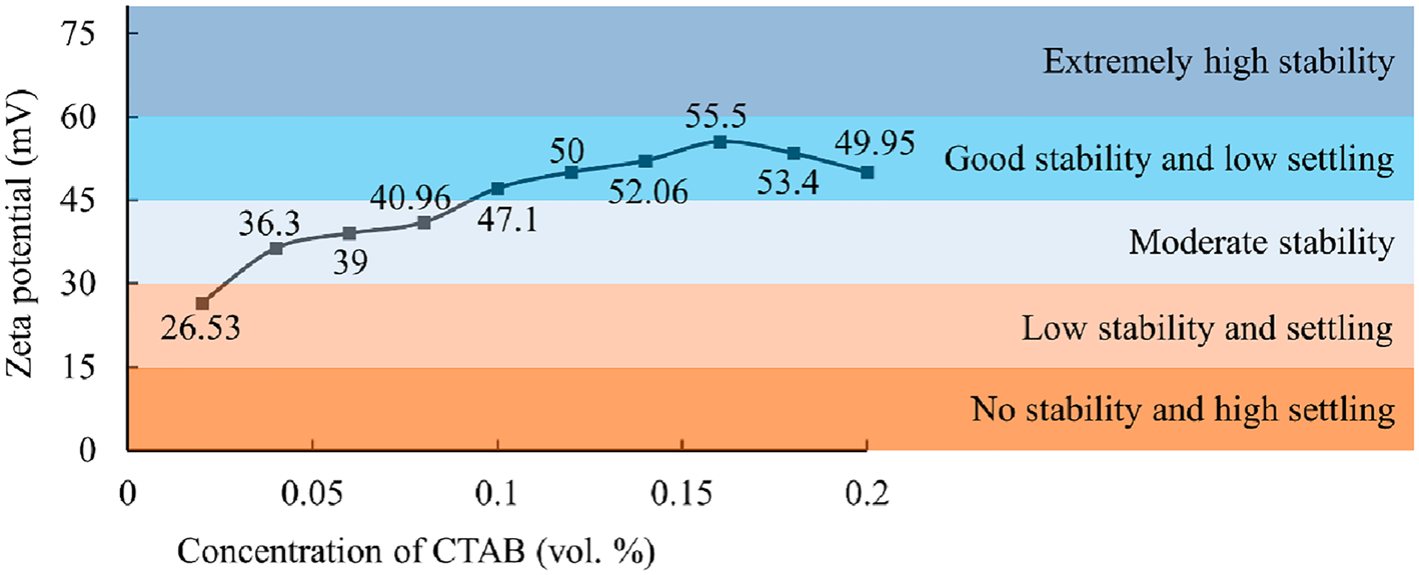
Zeta potential distribution of 0.025 vol.% Al12Mg17 nanofluids versus different concentrations of CTABs.

The zeta potential of 0.025 vol.% Al_12_Mg_17_ nanofluid at various CTAB concentrations is shown in Fig. 7. As depicted in Fig. 7, the zeta potential of the nanofluids falls within the range of 26.5–55.5 mV. Notably, the highest zeta potential, indicating the maximum stability, is observed for the nanofluid with a 0.16 vol.% CTAB concentration. Generally, zeta potential measurement follows the electrophoretic behavior monitoring to assess the stability of nanofluids^56^. A high zeta potential value corresponds to strong repulsive forces, which imply great stability^40^. In nanofluids, a high surface charge density causes considerable repulsive forces^57^. It is due to the fact that a low surfactant concentration cannot completely cover nanoparticles, consequently, a charge imbalance develops, which causes nanoparticles to aggregate and precipitate^58^.

There are some facts about the effect of surfactant concentration on nanofluid, which should be taken into account. On the one hand, increasing the concentration of surfactant desirably enhances the stability of nanofluid. On the other hand, surfactants undesirably weaken the heat conduction among base fluid and nanoparticles^59^. Also, with strong charge-stabilized dispersion, which results from higher surfactant concentration, the scattering intensity of DLS tests reduces^60^. Consequently, the concentration of 0.1 vol.% of CTAB appears to be a suitable choice for investigating the effect of 0.025 vol.% Al_12_Mg_17_ nanoparticle in DI water, since it not only provides a good stability range (Fig. 7) of zeta potential, but also it is the lowest possible concentration of surfactant to achieve a good stability.

### Particle size distribution

**Figure 8:**
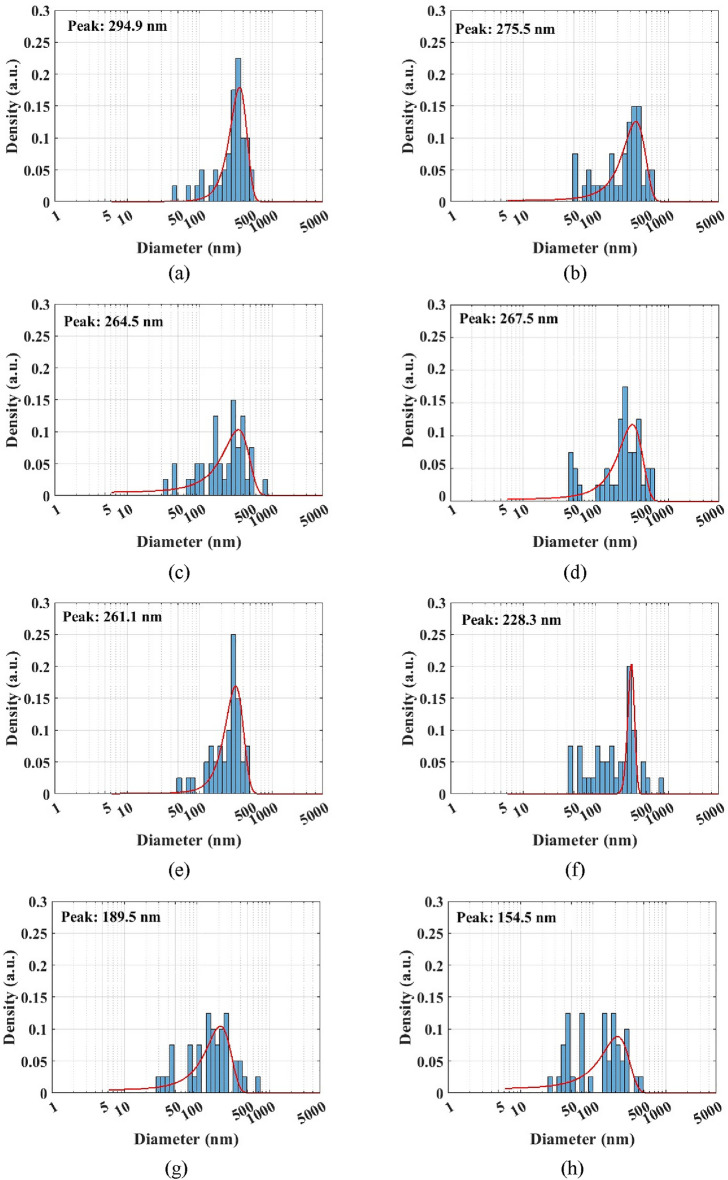

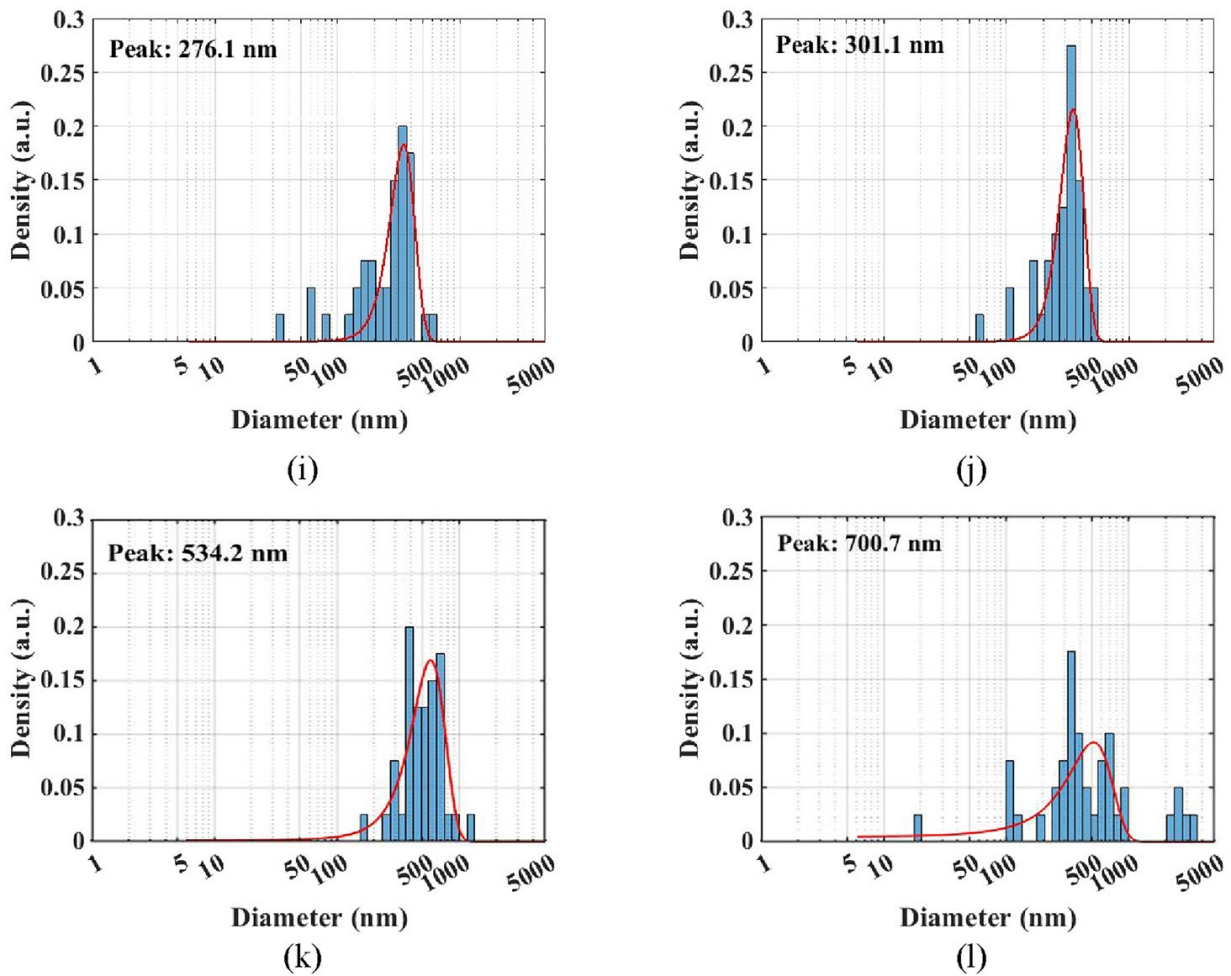
PSD of 0.025 vol.% Al_12_Mg_17_ nanofluid with 0.1 vol.% CTAB after ultrasonication periods of (**a**) 15 min, (**b**) 30 min, (**c**) 45 min, (**d**) 60 min, (**e**) 75 min, (**f**) 90 min, (**g**) 105 min, (**h**) 120 min, (**i**) 135 min, (**j**) 150 min, (**k**) 165 min, and (**l**) 180 min.

PSD for 0.025 vol.% Al_12_Mg_17_ nanofluid at different ultrasonication periods is shown in Fig. 8. The TEM results illustrated that Al_12_Mg_17_ nanoparticles adhered to one another and formed large clusters, which should be broken apart using an ultrasonic wave. Figure 8a represents the PSD for Al_12_Mg_17_ nanofluid after 15 min of ultrasonication, with a peak of 295 nm. Figure 8b–e show the PSD after 30, 45, 60 and 75 min of ultrasonication, respectively, which indicate that nanoparticles’ sizes remained noticeably unchanged. Moreover, Fig. 8f represents that the peak of PSD for Al_12_Mg_17_ nanoparticle decreases to 228 nm after 90 min of ultrasonication, while Fig. 8g,h show that when the ultrasonication period was increased to 105 min and 120 min, the peak of PSD decreased to 189 nm and 154 nm, respectively. As demonstrated in Fig. 8h, the optimum ultrasonication period is about 120 min in which the peak of PSD reaches to 154 nm. It should be noted that after two hours, the peak of PSD grew with increasing ultrasonication period, reaching to a value of 276 nm for an ultrasonication period of 135 min, and finally shifting to 700 nm for an ultrasonication period of 180 min (Fig. 8i–l).

**Figure 9:**
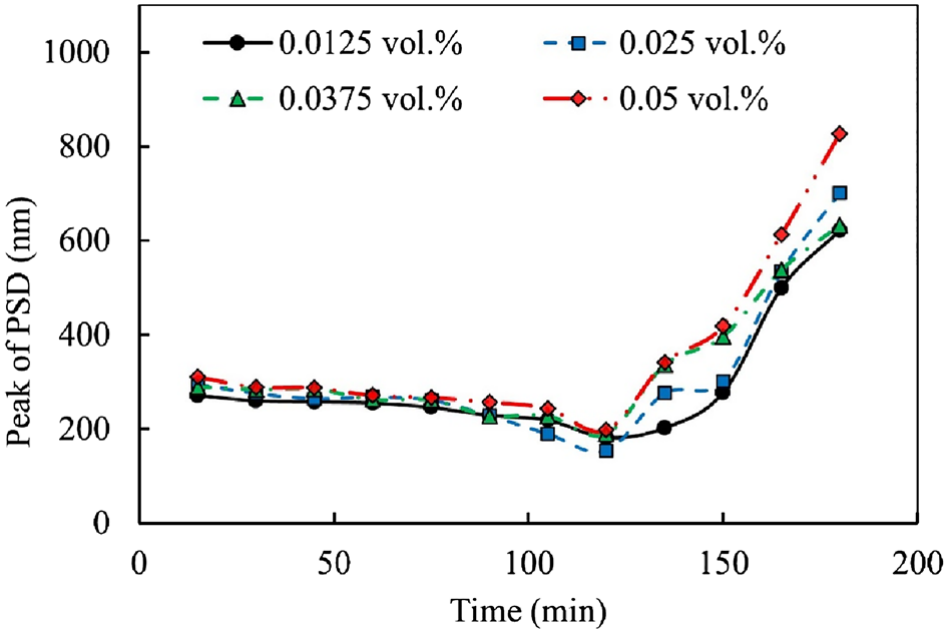
PSD peaks of Al_12_Mg_17_ nanofluid versus ultrasonication time at different concentrations of nanoparticles.

Figure 9 illustrates the peak of PSD for various concentrations of Al_12_Mg_17_ nanofluid (0.0125, 0.025, 0.0375, and 0.05 vol.%) with respect to ultrasonication time. The results indicate that after 120 min of ultrasonication, the PSD peaks for 0.0125, 0.025, 0.0375, and 0.05 vol.% Al_12_Mg_17_ nanofluid are 182.22, 154.5, 189.21, and 197.82 nm, respectively. Moreover, it is observed that in most cases, the PSD peaks tend to increase with an increase in the nanoparticles’ concentration. This phenomenon can be attributed to the presence of attractive interactions, such as van der Waals and depletion effects, which decrease with higher nanoparticle concentrations. In addition, hydrodynamic radius has an inverse relation with diffusion coefficient thus with an increase in the concentration the hydrodynamic radius increases.

Performing DLS measurements and analyses present certain challenges due to light absorption by the particle suspension. While lowering the particle concentration can reduce light absorption, higher concentrations yield better scattering intensity. Thus, striking a balance within the desirable concentration range can be challenging^60^.

Nanoclusters with larger sizes exhibit a greater tendency to undergo settling or precipitation. In contrast, the enhanced Brownian motion and stability of nanofluid can be attributed to nanoclusters with smaller sizes or nanoparticles, which allow for free movement within the base fluids^61^. In fact, the electro-kinetic properties and high surface charge density on the nanoparticles or nanoclusters determine the quality of dispersion in the cluster nanofluid. The stability of nanofluids is primarily influenced by the interactions between electrostatic repulsion and Vander Waals attraction energies among the nanoclusters in suspension, particularly in the presence of surfactants^27^.

Ultrasound sonication is a kind of vibration which provides the nanoparticle with a needed energy to be released from the force which holds it in place. Indeed, the energy that is applied during sonication in the nanofluid facilitates the movement of the nanoparticles. The nanoparticle cannot escape from the constriction force within the clusters if nanofluids do not obtain enough energy. On the other hand, the cluster collides with other clusters more frequently if too much energy is expended for moving it. Therefore, each cluster would be more likely to entangle with and interact with other clusters, which would result in the formation of larger clusters^62^. Consequently, it is important to determine the optimum ultrasonication period for the nanofluids. This quantity depends on the type of nanoparticle, the type of ultrasound sonication, the ultrasound’s power^51^, and the ultrasound’s pulse^63^. For example, it has been discovered that ultrasonic horn/probe devices are considerably more successful at dissolving the clusters rather than ultrasonic bath devices^64^. Continuous pulses, as another crucial factor affecting the dispersion of nanoparticles in fluid, can break up clusters into smaller pieces and the nanoparticles size distribution in the nanofluid becomes more uniform. Discontinuous pulses, however, are unable to completely disperse the clusters, and some sizeable aggregates can still be found in the nanofluid^63^. As mentioned above, the ultrasound’s power depends on the amount of energy which is needed for disintegrating the clusters to their constituent particles. Furthermore, for controlling the ultrasound’s power it should be considered that receiving too much energy can cause nanoparticles to start re-agglomeration^65^.

### Microstructural characterizations

**Figure 10:**
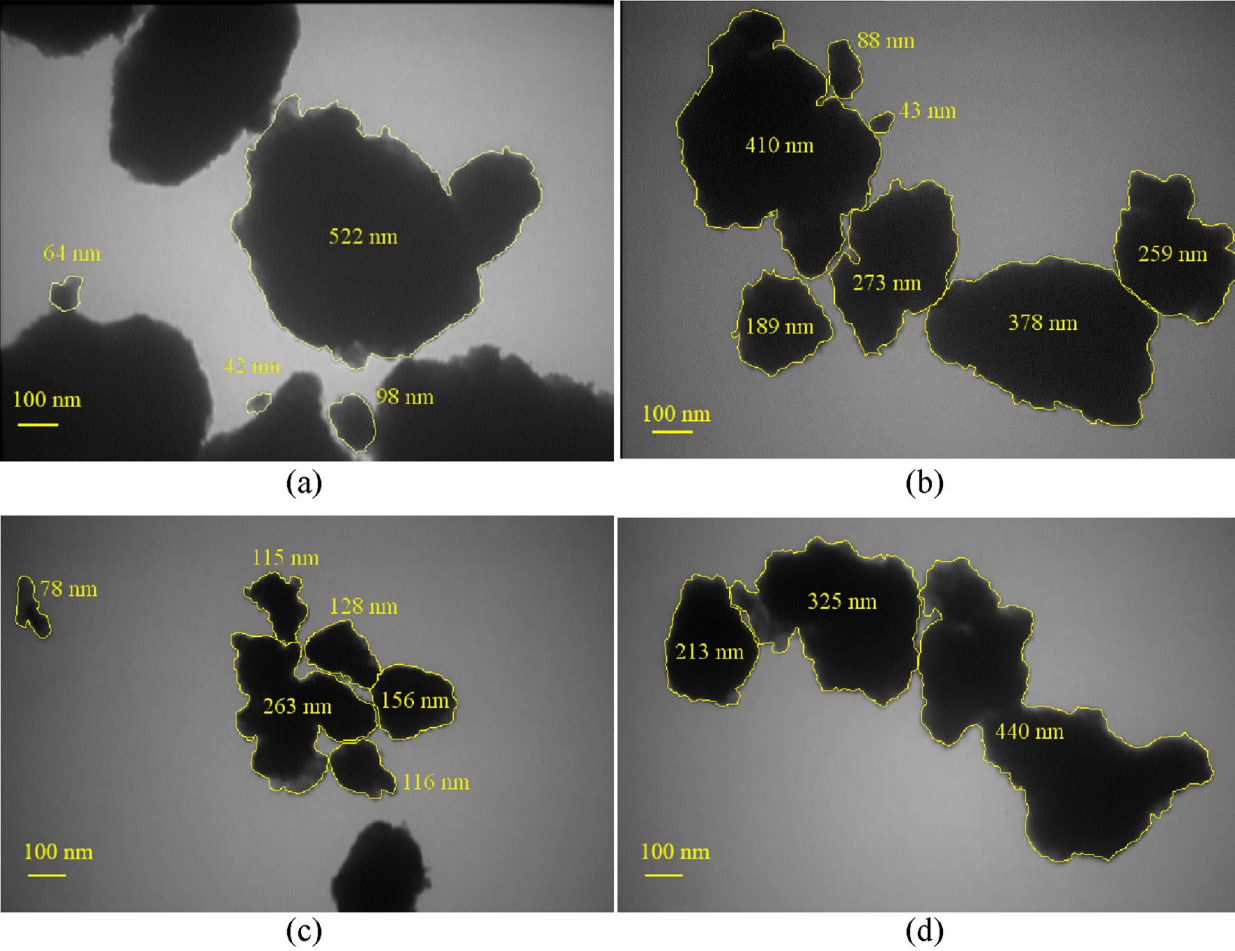
TEM results for the 0.025 vol.% Al_12_Mg_17_ nanoparticles in DI water with 0.1 vol.% CTAB. A view of cluster size distribution (**a**, **b**) after 15 min of ultrasonication, and (**c**, **d**) after 120 min of ultrasonication.

A drop of nanofluid was placed on a carbon grid for TEM scanning after the preparation of the nanofluid. In order to do an accurate analysis of both cluster size and nanofluid morphology and analyze the PSD, we took advantage of a bright-field TEM micrograph of the 0.025 vol.% Al_12_Mg_17_ nanofluid with 0.1 vol.% CTAB at various ultrasonication periods, which is depicted in Fig. 10. Figure 10a,b show the range of circular diameter of clusters after 15 min of ultrasonication. It should be noted that although the clusters are spherical or nearly spherical, as seen in Fig. 10a, their geometry is unknown, and it is estimated that their sizes are between 42 and 522 nm, and the average circular diameter of clusters is 181.5 nm. Moreover, according to Fig. 10b, the circular diameter of clusters of Al_12_Mg_17_ nanoparticles exhibits non-uniformity with cluster sizes ranging from 43 to 410 nm, and the average circular diameter of clusters is 234 nm.

The TEM results after 120 min of ultrasonication are presented in Fig. 10c,d which represent that the circular diameter of clusters is still non-uniform. Figure 10c, show that the circular diameter of clusters is between 78 and 263 nm, and the average of circular diameter of clusters is 142 nm. Besides, Fig. 10d, demonstrate that the circular diameter of clusters is between 213 and 440 nm and the average of circular diameter of clusters is 326 nm.

Furthermore, the PSD results demonstrated that they are not uniform, as seen in Fig. 8. It’s worth mentioning that after 15 min of ultrasonication, the PSD measurements revealed a range of clusters sizes between less than 50 to more than 500 nm (Fig. 8a). Additionally, the PSD after 120 min of ultrasonication, depicted in Fig. 8g, demonstrates that the particle sizes decrease in comparison with the PSD results after 15 min of ultrasonication, while the particle size is not uniform. In order to analyze PSD with TEM result, both of them demonstrate non-uniformity in clusters size after 15 and 120 min ultrasonication. Although the circular diameter of clusters can be measured through TEM microscopy, this method does not demonstrate the statistical perspective and therefor is unable to examine the optimum ultrasonication. In contrast, PSD result show the acceptable statistical perspective to investigate the optimum ultrasonication.

### HTC measurement, using beam displacement

**Figure 11:**
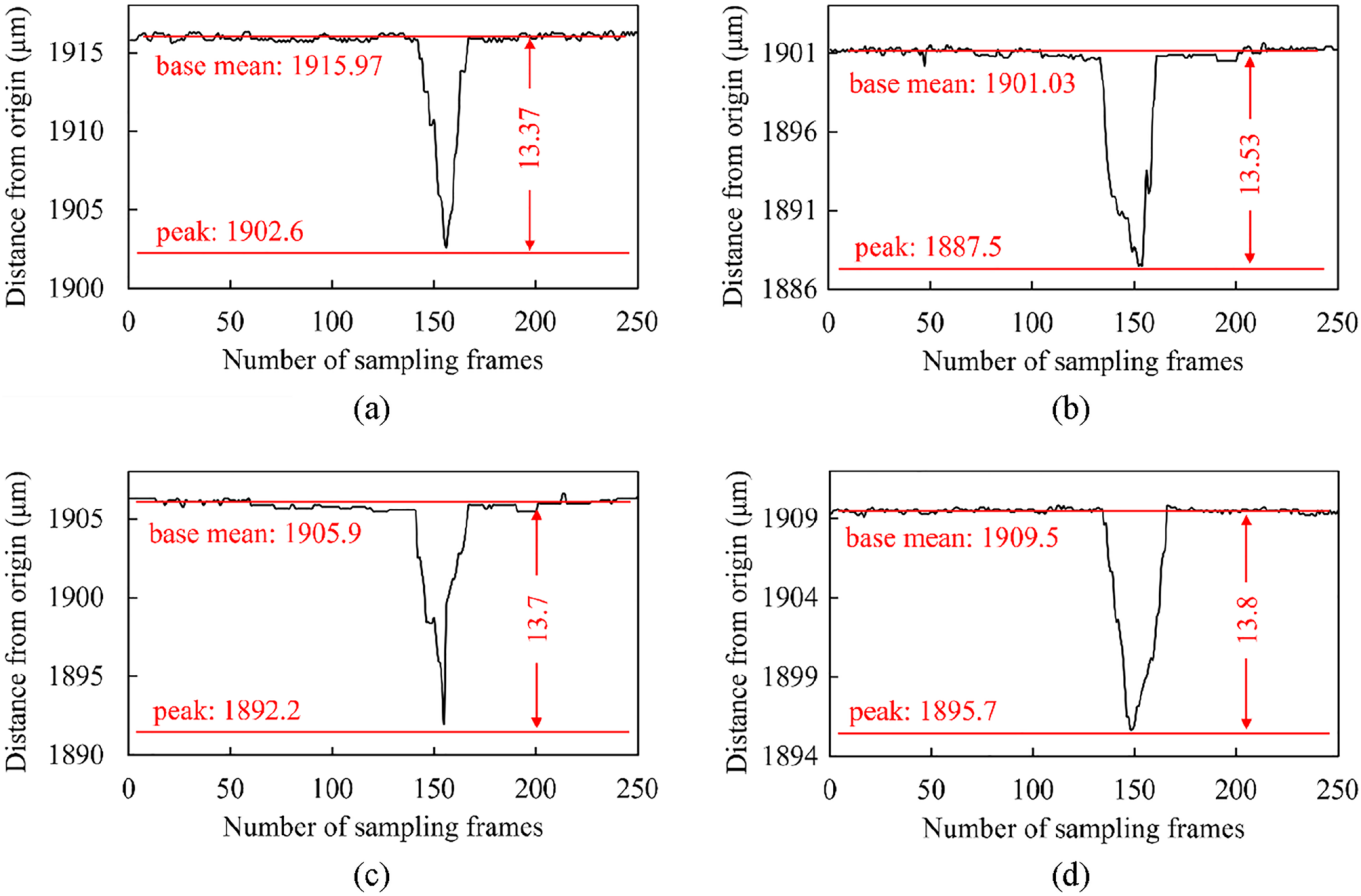
Beam displacement results for different concentrations of Al_12_Mg_17_ nanofluid at (**a**) 0.0125, (**b**) 0.025, (**c**) 0.0375, and (**d**) 0.05 vol.%.

As previously described, the beam displacement approach is used to determine the HTC of the Al_12_Mg_17_ nanofluid in three steps. The deviation of the beam, which is due to the thermal shock, is shown in Fig. 11. This figure shows the beam displacement measurements for various concentrations of Al_12_Mg_17_ nanoparticles versus the number of sampling frames. As shown in Fig. 11, beam displacements of 13.37, 13.53, 13.7, and 13.8 $\mathrm{\mu m}$ were measured for 0.0125, 0.025, 0.0375, and 0.05 vol.% Al_12_Mg_17_ nanofluids, respectively. Beam displacements were calculated as the difference between the average value of the beam-spot coordinate in the steady state and the displacement peak.

**Figure 12:**
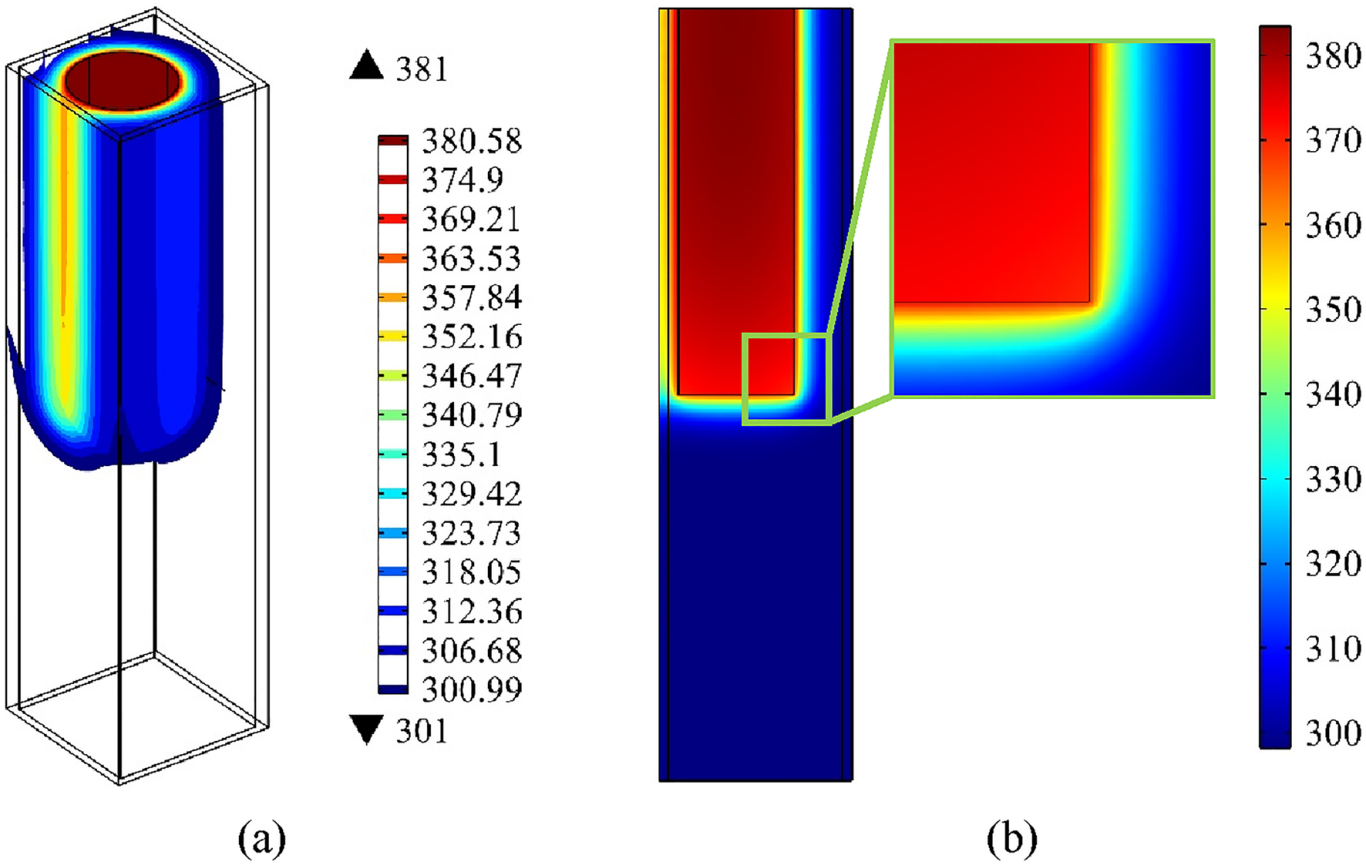
(**a**) Isothermal contour of the domain, and (**b**) temperature distribution on a cut plane parallel to xz-plane at y = 5 mm for the nanofluid’s HTC of 0.8 $\mathrm{W}/(\mathrm{m}.\mathrm{K})$ after 5 s.

As the outcome of the second step, a line corresponding to the beam path was employed in order to numerically analyze temperature variations. Figure 12 shows the thermal variations through the domain for the HTC of 0.8 $\mathrm{W}/(\mathrm{m K})$ after 5 s. Figure 12a,b show the isothermal contour and the temperature variation on a cut plane across the center of the heater (at y = 5 mm). This figure indicates that after 5 s of heating, there is a temperature difference of approximately 70° close to the heater, which leads to the beam displacement.

Following the third step, after the comparison of the experimental results with the numerical simulations, the HTC of the nanofluid was obtained. Therefore, the results of the beam displacement method demonstrated that the HTC of Al_12_Mg_17_ nanofluid is 0.61 $\mathrm{W}/(\mathrm{m K})$ at a concentration of 0.0125 vol.% and at concentrations of 0.025, 0.0375, and 0.05 vol.% increases to 0.66, 0.73, and 0.8 $\mathrm{W}/(\mathrm{m K})$, respectively.

**Figure 13:**
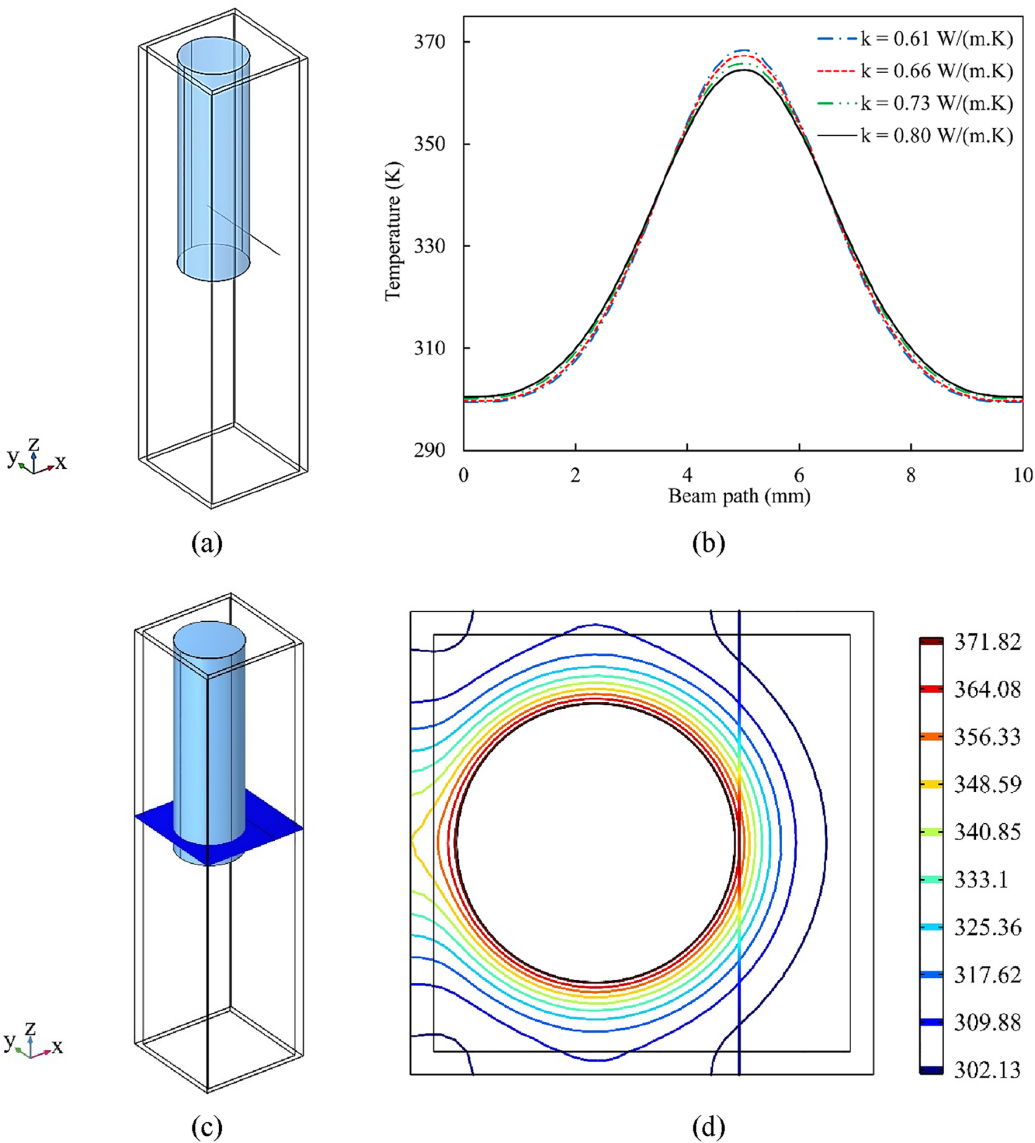
(**a**) A view of the beam path line (at $x=7.1 {\rm mm}, y=0-10 {\rm mm}, z = 22 {\rm mm}$), and (**b**) temperature variations on the beam path line after 5 s for different values of the nanofluid’s HTC. (**c**) A view of the cut plane parallel to xy-plane at z = 22 mm, and (**d**) temperature contour on the cut plane and the beam path line for $k=0.8\mathrm{ W}/(\mathrm{m} \mathrm{K})$ after 5 s.

The temperature variations on the beam path line (Fig. 13a) for different values of the nanofluid’s HTC are depicted in Fig. 13b. From this figure, it can be concluded that the temperature of a 0.05 vol.% Al_12_Mg_17_ nanofluid with an HTC of 0.8 W/(m K) is about 364 K, when locating at 100 microns from the heater after 5 s of heating. However, that of a 0.0125 vol.% Al_12_Mg_17_ nanofluid with an HTC value of 0.61 W/(m K) is approximately 368 K. Thus, heat transfer is improved by increasing the concentration of nanoparticles. Figure 13d shows the temperature contour of the nanofluid with an HTC of 0.8 $\mathrm{W}/(\mathrm{m K})$ on a cut plane parallel to xy-plane that crosses the beam path line (Fig. 13c). Given that fluids have a larger refractive index at lower temperatures, the nanofluid with the HTC of 0.8 $\mathrm{W}/(\mathrm{m K})$ has the lowest temperature changes among all and therefore has the largest displacement.

**Figure 14:**
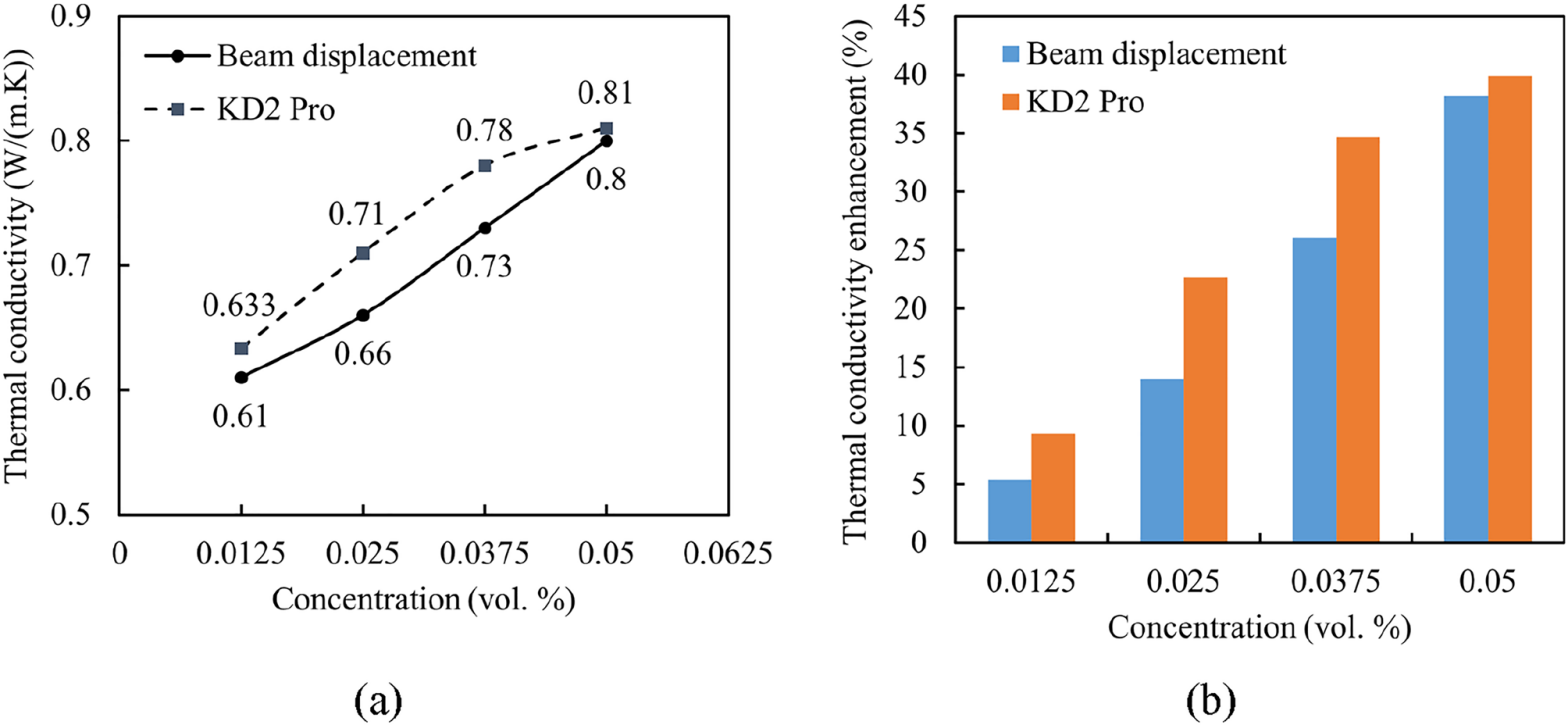
(**a**) Dependence of thermal conductivity of Al_12_Mg_17_ nanofluids on the concentration of nanoparticles with 0.1 vol.% CTAB. (**b**) HTC enhancement of the Al_12_Mg_17_ nanofluids compared to DI water.

In addition, the HTC measurements by the KD2 Pro apparatus were compared with the results, which were obtained from using the beam displacement approach. Figure 14 shows the HTC of the nanofluids measured using the KD2 Pro instrument at 25 °C and the beam displacement technique at various concentrations of Al_12_Mg_17_ nanoparticles. According to the KD2 Pro results, the HTC of the 0.0125 vol.% Al_12_Mg_17_ nanofluid is 0.633 $\mathrm{W}/(\mathrm{m K})$, which rises to 0.71, 0.78, and 0.81 $\mathrm{W}/(\mathrm{m K})$ by increasing the concentration of Al_12_Mg_17_ nanoparticles to 0.025, 0.0375, and 0.05 vol.%, respectively.

Based on the measured HTC values, the enhancements in the thermal conductivities were estimated relative to the base fluid as shown in Fig. 14b. The findings demonstrate that, compared to DI water with an HTC of 0.579 $\mathrm{W}/(\mathrm{m K})$, the thermal conductivity is higher when nanoparticles are present. With Al_12_Mg_17_ nanoparticles distributed in DI water at a concentration of 0.05 vol.%, the highest overall improvement in HTC of almost 40% and 38% was observed using KD2 Pro measurement and beam displacement method, respectively. On the other hand, the 0.0125 vol.% Al_12_Mg_17_ nanofluid exhibits the lowest magnitude of the HTC enhancement with a value of approximately 9% and 5% using KD2 Pro measurement and beam displacement method, respectively.

Different factors may contribute to the increased heat transfer in nanofluids, containing alloy nanoparticles, including Brownian motion, the cluster size, the aggregation of nanoparticles, the formation of a layer of fluid molecules close to the surfaces of the nanoparticles, the formation of nanoparticle complexes, and the collisions between clusters.^66^. It should be noted that thermal conductivity also depends on the crystal size, volume fraction of nanoparticles, and thermal characteristics of the solid suspension^67^.

Table 4 shows a comparison between the HTC enhancements of the understudied nanofluid and the results of two other studies. In the study of Paul et al.^20^, they noticed a 16% increase in HTC after dispersing 0.1 vol.% of Al_95_Zn_05_ nanoparticles in ethylene glycol. They asserted that the increased rate of heat transfer in nanofluids is attributed to the large specific surface area of the nanoparticles, the particle shape factor, liquid layering at the solid–liquid interface, clustering/aggregation, and the Brownian motion. Furthermore, the research conducted by Karthik et al.^24^ indicated that adding 0.1 vol.% Ni_65_Al_35_ nanoparticles boosted the HTC of nanofluid by 28%. They also showed that the Ni_65_Al_35_ intermetallic surface composition and the bulk stoichiometry had a small effect on the increase of nanofluids’ thermal conductivity, containing Ni_65_Al_35_ nanoparticles. Therefore, in the present study, Al_12_Mg_17_ nanoparticles distributed in DI water outperform Ni_65_Al_35_ and Al_95_Zn_05_ nanoparticles dispersed in water and ethylene glycol, respectively.

**Table 4:** A comparison among the HTC enhancements reported by different references and the current research.

Study	Base fluid	Nanoparticle	Concentration (vol.%)	HTC enhancement (%)
Paul et al.^20^	Ethylene glycol	Al_95_Zn_05_	0.1	16
Karthik et al.^24^	Water	Ni_65_Al_35_	0.1	28
Present study	Water	Al_12_Mg_17_	0.05	40

### Electrical conductivity enhancement

**Figure 15:**
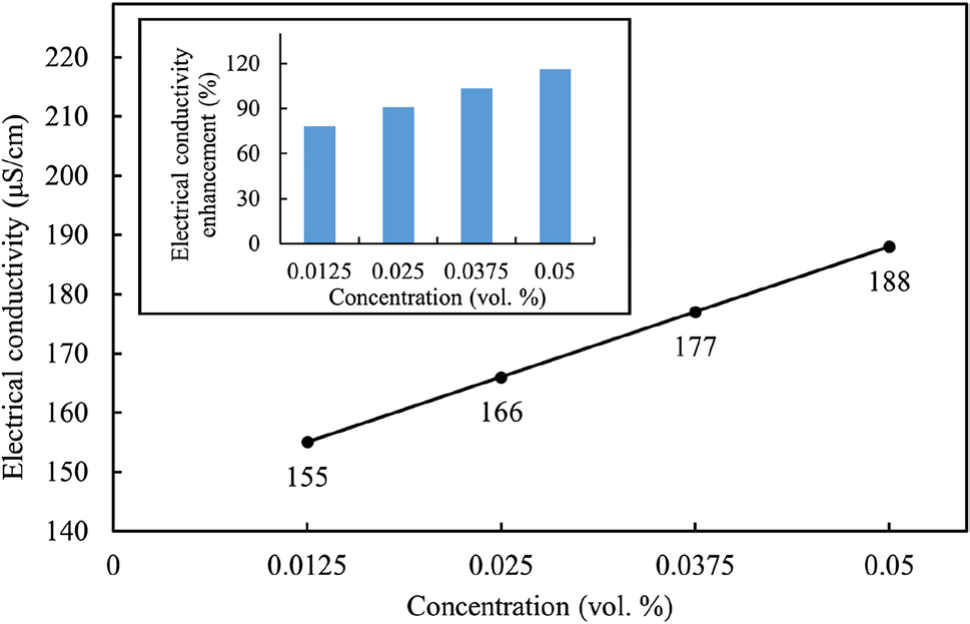
The effective electrical conductivity of Al12Mg17 nanofluid at various concentrations of nanoparticles with 0.1 vol.% CTAB at $25\mathrm{^\circ{\rm C} }$.

The results of the electrical conductivity measurements for the Al_12_Mg_17_ nanofluid at different concentrations of nanoparticles are shown in Fig. 15. In the absence of Al_12_Mg_17_ nanoparticles, a base fluid containing 0.1 vol.% CTAB has an electrical conductivity of 86 $\mathrm{\mu S}/\mathrm{cm}$. As shown in Fig. 15, the electrical conductivity of Al_12_Mg_17_ nanofluid increases linearly from 155 to 188 $\mathrm{\mu S}/\mathrm{cm}$ by enhancing the volume fraction from 0.0125 to 0.05 vol.%. The enhancements in the electrical conductivities of nanofluids were calculated relative to the base fluid containing 0.1 vol.% CTAB. The maximum enhancement in electrical conductivity of about 116% was observed for the 0.05 vol.%. Al_12_Mg_17_ nanofluid. The electrical conductivity of a nanofluid is associated with the ability of the charged ions inside the nanofluid to transport electrons. This might be due to the possible formation of an electrical double-layer on the surface of the dispersed nanoparticles^68^. The primary cause of the increase in electrical conductivity is the creation of surface charges caused by the polarization of nanoparticles when dispersed in the polar water. The dispersion of the nanoparticles alters the dielectric constant and density of the base fluid. Therefore, it seems reasonable that an increase in the electrical conductivity would follow a rise in the concentration of Al_12_Mg_17_ nanoparticles.

The electrical conductivity of suspension was first modelled by Maxwell. The model could also be applied to describe nanofluids as follows:

6 $$\frac{{\sigma }_{nf}}{{\sigma }_{0}}=1+ \frac{3(\alpha -1){\varphi }_{\upsilon }}{\left(\alpha +2\right)-(\alpha -1){\varphi }_{\upsilon }}$$

**Figure 16:**
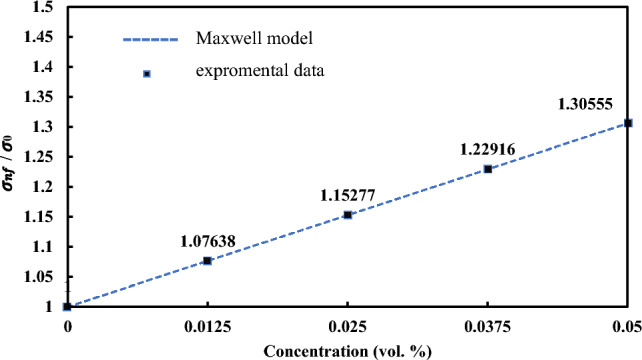
Fitting of the Maxwell model and a linear function related to Al_12_Mg_17_ nanofluid.

This model addresses the increase of electrical conductivity in spherical particle suspensions with a low vol.% in base fluid. In Eq. (6), α = (σp)/(σ0), σnf is the electrical conductivity of nanofluid, σ0 is the base fluid conductivity, and σp is the particle conductivity^69^ Fitting a linear function is the most effective method for the experimental data extracted from Al_12_Mg_17_ nanofluids since all the findings were linear. Figure 16 depicts the fitting of the Maxwell model and a linear function related to Al_12_Mg_17_ nanofluids. Maxwell’s static model predicts a linear relationship between electrical conductivity and concentration; however, it ignores Brownian motion, aggregation, and the electric double layer (EDL). Equation (7) represent a liner relation between electrical conductivity and concentration. related to Al_12_Mg_17_ nanofluids.

7 $$\frac{{\sigma }_{nf}}{{\sigma }_{0}}=1+ 6.111\varphi$$

## Conclusion

In this study, comprehensive experiments were conducted to determine the stability, optimum ultrasonication period, thermal conductivity, and electrical conductivity of the Al_12_Mg_17_ nanofluid. Our study can shed light on the potential of nanofluids as coolants in heat transfer devices such as solar collectors, automobile radiators, and electronic cooling systems. Our study addresses a crucial aspect which has received limited attention so far, namely the evolution of PSD with different ultrasonication periods to determine the optimal duration. By employing the DLS method in conjunction with TEM micrograph imaging, the study successfully characterized the PSD and confirmed its accuracy. The stability of the nanofluids was meticulously evaluated through visual observation and zeta potential measurements. Furthermore, the introduction of a novel beam displacement method, combined with numerical analysis, provided an innovative approach to measuring the HTC accurately. The utilization of optical methods allowed for swift data collection, enabling assessments prior to the emergence of bulk motion within the nanofluid caused by thermal fluctuations. To validate the HTC data obtained from the beam displacement method, a comparison with results from the reliable KD2 Pro apparatus was performed. Additionally, the electrical conductivity of the nanofluid was measured using the PCT 407 apparatus.

The attainment of proper stability of the nanofluid was accomplished through the utilization of CTAB as the most appropriate surfactant, within the concentration range of nanoparticles from 0.0125 to 0.05 vol.%. The findings of the investigation indicate that a duration of 2 h of ultrasonication yielded a maximum PSD of 154 nm, which was subsequently scrutinized via TEM imaging. The results of the zeta potential analysis demonstrate that adding 0.16 vol.% CTAB for 0.025 vol.% Al_12_Mg_17_ nanofluid results in the maximum stability. Furthermore, the observation method findings indicate that nanoparticles’ surface bears a negative charge. The highest HTC enhancement of 40% was achieved at a nanoparticle concentration of 0.05 vol.%, demonstrating the potential for substantial heat transfer improvements. Additionally, the electrical conductivity exhibited a remarkable linear increase, with the highest value of 188 $\mathrm{\mu S}/\mathrm{cm}$ attained at the same concentration of Al_12_Mg_17_ nanoparticles.

In conclusion, the findings of this study highlight the exceptional characteristics of Al_12_Mg_17_ nanofluid and its considerable potential for enhancing heat transfer performance. The novel beam displacement method introduced in this research offers a cutting-edge technique for accurate determination of HTC. These advancements contribute significantly to the ongoing exploration of nanofluids and pave the way for their practical implementation in various heat transfer applications, ultimately advancing the efficiency and sustainability of thermal systems.

## Data Availability

The datasets used and analyzed during the current study available from the corresponding author on reasonable request.
